# Adenovirus-Mediated Sensitization to the Cytotoxic Drugs Docetaxel and Mitoxantrone Is Dependent on Regulatory Domains in the E1ACR1 Gene-Region

**DOI:** 10.1371/journal.pone.0046617

**Published:** 2012-10-03

**Authors:** Enrique Miranda, Hector Maya Pineda, Daniel Öberg, Gioia Cherubini, Zita Garate, Nick R. Lemoine, Gunnel Halldén

**Affiliations:** Centre for Molecular Oncology, Barts Cancer Institute, Queen Mary University of London, London, United Kingdom; Ecole Normale Supérieure de Lyon, France

## Abstract

Oncolytic adenoviruses have shown promising efficacy in clinical trials targeting prostate cancers that frequently develop resistance to all current therapies. The replication-selective mutants AdΔΔ and *dl*922–947, defective in pRb-binding, have been demonstrated to synergise with the current standard of care, mitoxantrone and docetaxel, in prostate cancer models. While expression of the early viral E1A gene is essential for the enhanced cell killing, the specific E1A-regions required for the effects are unknown. Here, we demonstrate that replicating mutants deleted in small E1A-domains, binding pRb (*dl*1108), p300/CBP (*dl*1104) and p400/TRRAP or p21 (*dl*1102) sensitize human prostate cancer cells (PC-3, DU145, 22Rv1) to mitoxantrone and docetaxel. Through generation of non-replicating mutants, we demonstrate that the small E1A12S protein is sufficient to potently sensitize all prostate cancer cells to the drugs even in the absence of viral replication and the E1A transactivating domain, conserved region (CR) 3. Furthermore, the p300/CBP-binding domain in E1ACR1 is essential for drug-sensitisation in the absence (AdE1A1104) but not in the presence of the E1ACR3 (*dl*1104) domain. AdE1A1104 also failed to increase apoptosis and accumulation of cells in G2/M. All E1AΔCR2 mutants (AdE1A1108, *dl*922–947) and AdE1A1102 or *dl*1102 enhance cell killing to the same degree as wild type virus. In PC-3 xenografts *in vivo* the *dl*1102 mutant significantly prolongs time to tumor progression that is further enhanced in combination with docetaxel. Neither *dl*1102 nor *dl*1104 replicates in normal human epithelial cells (NHBE). These findings suggest that additional E1A-deletions might be included when developing more potent replication-selective oncolytic viruses, such as the AdΔCR2-mutants, to further enhance potency through synergistic cell killing in combination with current chemotherapeutics.

## Introduction

Several replication-selective oncolytic adenoviral mutants have been developed as potential therapies for the treatment of various cancers (virotherapy) including prostate cancer [1], [2], [3]. Prostate cancer is a leading cause of cancer-related morbidity and mortality in aging men globally with development of resistance to all currently available therapies including anti-androgens and cytotoxic drugs. Therefore, therapeutics with different mechanisms of action are urgently needed.

Virotherapy is one promising strategy to target treatment-resistant prostate cancers and several mutants have been evaluated in clinical trials for this malignancy [2]. The androgen receptor (AR) is active in the majority of prostate tumors which enabled the generation of adenoviral mutants with replication controlled by AR response elements (AREs) to prevent replication in non-prostate tissue [4]. In addition to altered AR-activity, prostate cancers frequently present with genetic alterations in cell cycle and cell death pathways including Ras/Raf/MEK/ERK, JAK/STAT and PI3K/AKT or deregulated pRb, p16, p53, PTEN, Bcl2 and related factors [5], [6], [7], [8]. These alterations have also been exploited for development of oncolytic adenoviruses since they complement and support replication of mutants deleted in the genes regulating the same pathways, while replication in normal tissue cannot proceed. One example is the modified *dl*1520 mutant Ad5-CD/TK*rep* [9], [10], which has the E1B55K gene deleted with replication complemented by non-functional p53, and mRNA-export and/or translation in cancer cells [11], [12]. Ad5-CD/TK*rep* also expresses the chimeric suicide gene CD/HSV-TK and was reported to have long-term benefits in patients with localized disease in combination with the prodrugs 5-fluorocytosine (5-FC) and ganciclovir (GCV) or radiotherapy [13]. An optimized version, Ad5-yCD/*mut*TK_SR39_ *rep*-ADP is currently being evaluated in a phase II/III randomized clinical trial in combination with chemo- and radio- therapies (NCT00583492: www.clinicaltrials.gov) [14].

Even though clinical safety of replication-selective adenoviruses has been demonstrated in hundreds of patients, efficacy was only reported in combination with other cytotoxic factors including cisplatin, 5-fluorouracil (5-FU), gemcitabine or radiation [1], [15]. Preclinical studies also demonstrate that several recently developed E1ACR2-deleted mutants such as AdΔCR2, AdΔΔ and AdΔ24, complemented by deregulated pRb/cell cycle pathways, have significantly higher efficacy in combination with various cytotoxic drugs in prostate cancer models [16], [17], [18], [19], [20]. Furthermore, adenoviruses can infect and kill both proliferating and non-proliferating tumor cells, an important consideration in the treatment of prostate cancers that are often slow growing.

Numerous studies have convincingly demonstrated that adenoviruses can interact synergistically with cytotoxic drugs to enhance cancer cell killing, but the cellular mechanisms involved in the responses are poorly understood. Expression of the early viral E1A proteins in the absence of other viral genes and replication is sufficient to induce apoptosis in cancer and normal cells and extensive data implicate a role also in chemosensitization [21], [22], [23], [24], [25], [26], [27], [28], [29]. The E1A transcript is differentially spliced to generate five proteins; 13S, 12S, 11S, 10S and 9S that peak at different time-points after infection. Numerous cellular proteins bind to E1A mainly through three conserved regions (CR1–3) each associated with specific proteins and functions [11], [30], [31], [32]. The CR3 region is only present in E1A13S and is essential for activation of viral and cellular genes. E1A-mediated sensitization to cytotoxic drugs has been reported for the two major E1A proteins, 12S and 13S, and does not appear to depend on E1ACR3-mediated transcriptional activation [25], [26], [27], [28]. It is not clear whether E1ACR2-binding to pRb plays a role in drug-sensitization since both increased and decreased cell killing has been reported with ΔCR2 mutants [16], [19], [28], [29], [33], [34]. The E1ACR1 and E1A N-terminal domains were reported to contribute to drug-sensitization; deletion of CR1 partially impaired sensitisation to adriamycin and deletion of the N-terminus prevented sensitization [28], [29]. Some important functions of the N-terminal and CR1 domains are binding of histone acetyltransferases (HATs) and cell cycle regulators such as p300/CBP, p400, PCaF, TRRAP and p21, to alter cellular transcriptional activation and repression, mRNA translation and protein stability, in favour of viral amplification [11], [30], [31]

In this study we screened a panel of replicating mutants with small E1A-deletions previously demonstrated to be defective in binding to pRb (*dl*1108, *dl*922–947), p300 and p400 (*dl*1101), p300/CBP (*dl*1104), and p400 and p21 (*dl*1102) [24], [31], [35], [36], [37], to explore whether the specific E1A gene regions that bind to these and other cellular factors are essential for sensitization to drugs currently used in the clinic for prostate cancer: mitoxantrone, a topoisomerase inhibitor, and docetaxel, a microtubule-interfering drug. Replication-defective mutants with the corresponding deletions in the small E1A12S protein were generated to further explore E1A-mediated effects in the absence of E1ACR3-mediated transcriptional activation or viral replication. We demonstrate that expression of the small E1A12S protein alone was sufficient to sensitise prostate cancer cells to both drugs. E1A12S-mutants with deletions in the p400/p21- (AdE1A1102) or pRb- (AdE1A1108) binding regions were highly potent and synergised with the drugs. In contrast, deletion of the p300/CBP-binding site (AdE1A1104) severely attenuated efficacy and sensitization while the corresponding replicating E1A13S mutant (*dl*1104) was less severely attentuated. Neither *dl*1102 nor *dl*1104 sensitized normal prostate (PrEC) and bronchial (NHBE) epithelial cells to the drugs, and replication was greatly attenuated. In a prostate cancer *in vivo* xenograft model (PC-3), tumor progression was significantly inhibited with *dl*1102, both alone and in combination with docetaxel. Our data suggest that future developments of oncolytic adenoviruses may include additional deletions in the region preceding the p300/CBP binding site in the E1ACR1 domain but not within CR1, to improve on selectivity, decrease toxicity to normal cells and potently synergise with chemotherapeutics to kill cancer cells only.

## Results

### Replicating adenoviral mutants with small deletions in the E1A-region have higher potency than the E1B55K-deleted dl1520 mutant in the human PC-3 and DU145 prostate cancer cell lines

**Figure 1:**
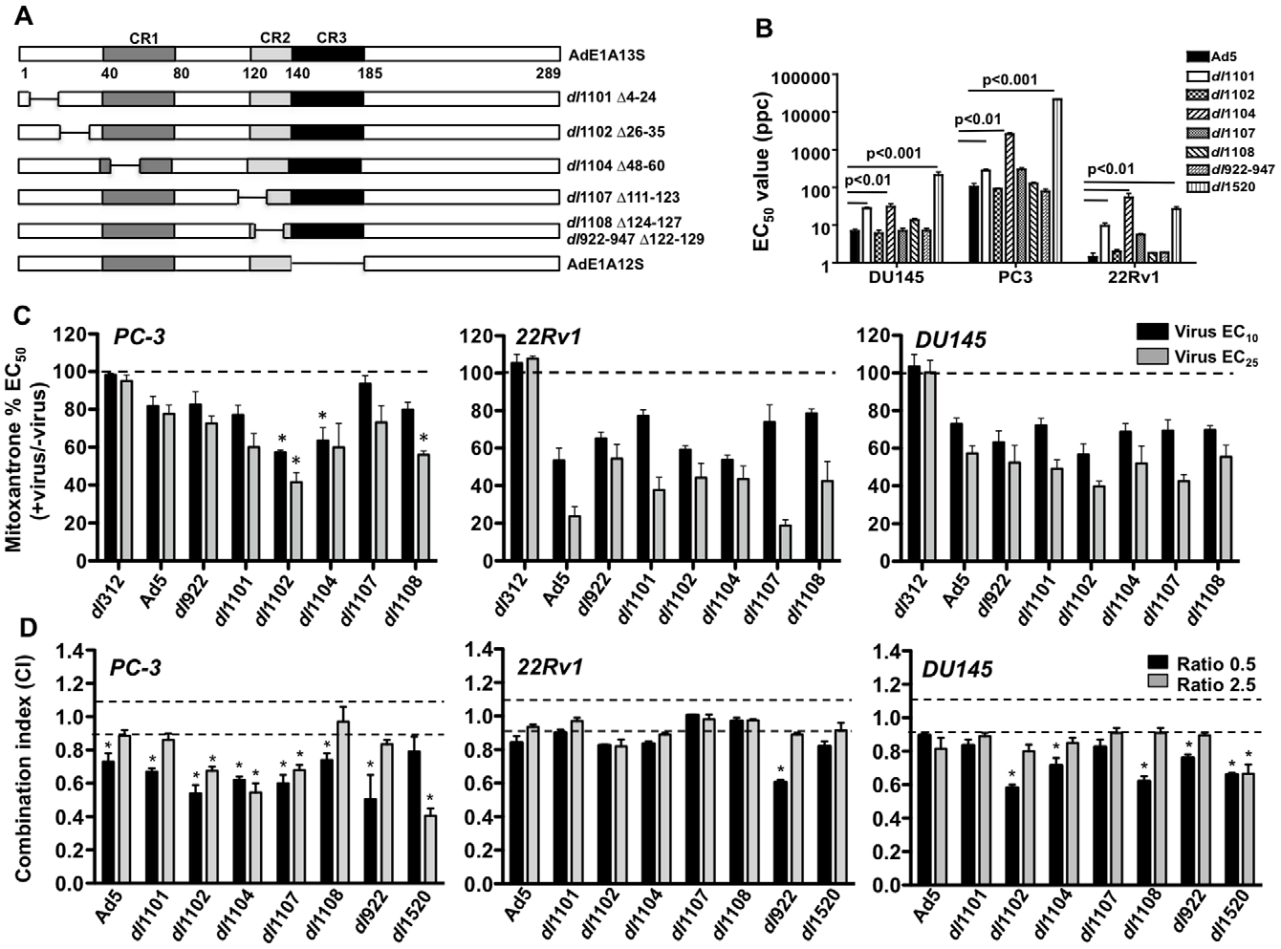
Potent cell killing of prostate cancer cell lines by replicating E1A-deletion mutants in combination with mitoxantrone. A) The replicating viruses used in the study had intact E1A-region (E1A13S) except for the indicated deletions. The replication-defective mutants were based on the E1A12S construct with the same deletions as in the replicating viruses; AdE1A1102 (Δ26–35), AdE1A1104 (Δ48–60), AdE1A1108 (Δ124–127), in addition to deletion of the CR3-region, responsible for viral transcriptional activity. B) EC_50_ values for the replicating mutants were determined from dose-response curves and presented as averages ± SD, n = 3. Significantly different values compared to Ad5 are indicated. C) Sensitization of the human PC-3, 22Rv1 and DU145 cells to mitoxantrone by fixed doses of each virus at EC_10_ and EC_25_. Data presented as percentages of mitoxantrone EC_50_ values in each cell line, averages ± SD, n = 3. Statistical analysis by 1-way Anova, *p<0.05 for drug EC_50_ values that were significantly lower than the corresponding Ad5 values. The *dl*312 (ΔE1A) non-replicating virus served as negative control. D) Graphic representation of combination indexes (CI) generated from synergy studies with mitoxantrone in combination with each replicating viral mutant at two constant ratios 0.5 and 2.5 viral particles per cell (ppc)/nM drug. Synergistic interactions are represented by CI≤0.9, antagonism by CI≥1.1 and additive effects by 0.9<CI<1.1, averages ± SEM, n = 3–5, *p<0.05 by t-test compared to the theoretical additive values.

Replicating mutants that are defective in binding to p300/CBP (*dl*1101, *dl*1104), p400/p21 (*dl*1101, *dl*1102), pRb, p130 and p170 (*dl*922–947, *dl*1108), or pRb and p130 (*dl*1107) [35], [36], [37], [38], were evaluated for cytotoxicity in human prostate cancer cells (Fig. 1A). The PC-3 cells were highly insensitive with EC_50_ values for Ad5 wild type virus of 104±18 ppc while 22Rv1 and DU145 cells were at least ten times more sensitive at 1.4±0.6 ppc and 6.9±1.3 ppc respectively (Fig. 1B). Sensitivity to each mutant varied, with significantly lower potency for viruses with deleted p300/CBP-binding domains (*dl*1101 and *dl*1104) (p<0.01). However, all mutants had higher potency than the attenuated *dl*1520 virus deleted in the E1B55K gene, one of the most extensively clinically evaluated oncolytic mutant (*a.k.a.* ONYX-015). In the 22Rv1 cells the *dl*1104 mutant was slightly less efficacious than *dl*1520. The murine prostate cancer cell lines TRAMPC and RM1 were significantly less sensitive to all viruses than the human cells with EC_50_ values for Ad5 at 7500±1900 and 2700±600 ppc respectively (Supporting Fig. S1). Interestingly, the *dl*1101 and *dl*1104 were also among the least potent mutants in these cells while *dl*1520 was more potent in the TRAMPC cells. The virus-insensitive PC-3 cells were also highly insensitive to the chemotherapeutics currently used for late-stage prostate cancer, mitoxantrone and docetaxel (p<0.001 and p<0.05 respectively) compared to DU145 and 22Rv1 (Supporting Fig. S2A). Both TRAMPC and RM1 were as sensitive to mitoxantrone as the DU145 and 22Rv1 cells but less sensitive to docetaxel (Supporting Fig. S2B). The differences in potency between mutants were not caused by variations in viral activity since all replicating mutants had vp/pfu ratios of 10–20 (Supporting Table S1).

### The replicating E1A-deletion mutants enhance cytotoxic drug-induced cell killing

We previously demonstrated synergistic anti-tumor efficacy for Ad5, *dl*1520 and E1ACR-deleted mutants with mitoxantrone or docetaxel in prostate cancer models [16], [19], [39]. To explore whether mutants with the small E1A-deletions evaluated above (Fig. 1A–B) could further improve on drug-induced cell killing, low doses (EC_10_ and EC_25_) of each deletion-mutant were tested in combination with mitoxantrone. We found that all mutants sensitized both virus- and mitoxantrone-insensitive (PC-3) and virus- and mitoxantrone-sensitive (22Rv1 and DU145) cells (Fig. 1C). In the PC-3 cells, only *dl*1102 was significantly more efficacious (p<0.05) than Ad5 at both doses while other mutants sensitized the cells to similar levels as Ad5 or slightly more at one dose (*e.g. dl*1108). In 22Rv1 and DU145 cells potent sensitization was observed with all mutants to similar levels as with Ad5. Interestingly, the murine virus-insensitive and mitoxantrone-sensitive TRAMPC cells were sensitized with all mutants and *dl*1101 significantly decreased the mitoxantrone EC_50_ value compared to Ad5 (p<0.05) (Supporting Fig. S3A). The non-replicating E1A-deleted *dl*312 mutant had no effect on drug-induced cell killing in any cell line (Fig. 1C). Several mutants also induced synergistic cell killing, determined by combination indexes (CI) at two constant ratios (Fig. 1D). In PC-3 cells, the synergy was significant with all mutants at one or both ratios (CI≤0.9; p<0.05 compared to the theoretical additive value 0.9<CI<1.1). In DU145 cells, significant synergy was observed with *dl*1102, *dl*1104, *dl*1108, *dl*922–947 and *dl*1520 at one or two ratios (p<0.05) and in 22Rv1 cells only with the *dl*922–947 mutant at one condition. A trend towards synergy was also seen in the TRAMPC cells with significant effects with *dl*922–947 and *dl*1520 (p<0.05) (Supporting Fig. S3B). Similar synergistic cell killing was determined in combination with docetaxel, again with the greatest effects in PC-3 cells and the least in 22Rv1 cells (data not shown). We conclude that the highly virus- and drug-resistant PC-3 cells were most effectively sensitized to the combination treatments with all mutants. The role of specific E1A-regions could not be conclusively determined with this strategy since viral replication significantly contributed to the cell killing in the human prostate cancer cells. Furthermore, 22Rv1 and DU145 cells support adenoviral replication more efficiently than PC-3 cells [16], [19].

### Expression of the E1A12S region alone causes strong synergistic cell killing in combination with mitoxantrone and docetaxel

**Figure 2:**
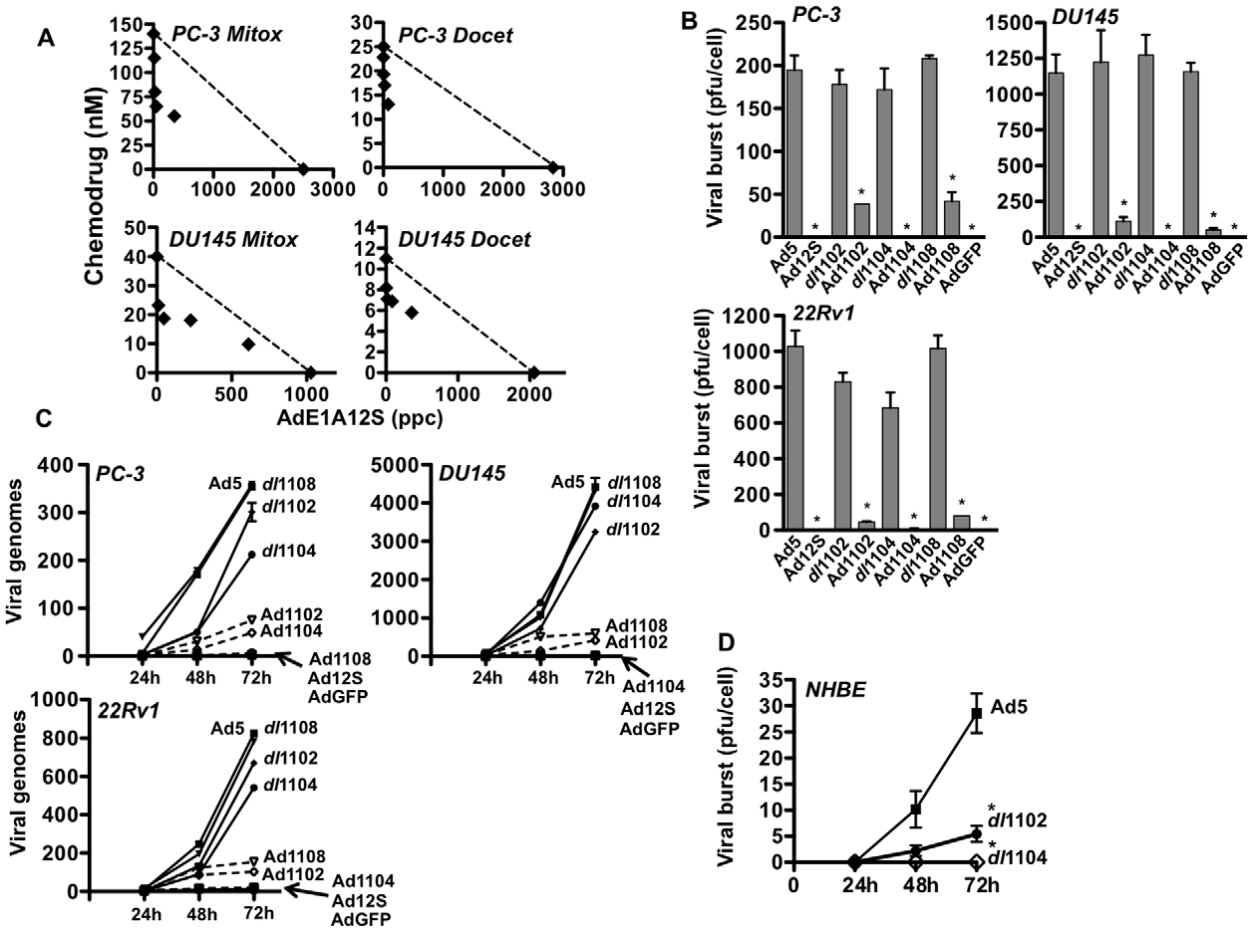
Synergistic cell killing with a replication-defective virus expressing the small AdE1A12S protein, in combination with cytotoxic drugs. A) Isobolograms generated from EC_50_ values for combinations of the AdE1A12S mutant with mitoxantrone (Mit) or docetaxel (Doc) at four constant ratios (0.5. 2.5, 12.5 and 62.5 ppc/nM drug) in PC-3 and DU145 cells. The straight lines represent the theoretical values for additive effects and points below the line synergistic cell killing, one representative study (n = 3–4). B) Characterization of replication of the AdE1A12S, AdE1A1102, AdE1A1108 and AdE1A1104 mutants in PC-3, DU145 and 22Rv1 cells. Levels of viral replication determined by the limiting dilution assay (TCID_50_) for replicating and replication-defective mutants with identical E1A-deletions except for the additional deletion of the CR3-domain in E1A12S. Cells were infected with each mutant at 100 ppc and harvested 72 h later, averages ±SD, n≥3. The non-replicating AdGFP mutant was used as a control in all assays, *p<0.001 for the replicating compared to the corresponding replication-defective mutant (t-test). C) qPCR analysis of cells infected as described for the replication assays and harvested 24, 48 and 72 h later. Total copy number at each time point was normalised to the copy numbers detected 3 h after infection in 10 ng of total DNA, averages ± SEM, n = 2–3. D) Viral replication in normal human primary bronchial epithelial cells (NHBE) determined by TCID_50_ for Ad5wt, *dl*1102 and *dl*1104 mutants infected at 100 ppc, n = 3, *p<0.005.

To investigate whether E1A expression alone, without contribution from additional viral genes and viral replication, could sensitize prostate cancer cells to the cytotoxic drugs, an expression plasmid was constructed encoding only the small E1A12S (ΔCR3) cDNA under control of the CMV promoter (Fig. 1A). Transient E1A12S expression resulted in sensitization to both mitoxantrone and docetaxel compared to the corresponding GFP-expressing control vector in PC-3, DU145 and 22Rv1 cells (Table 1). Although, the transfection conditions caused low levels of cell death the drug EC_50_ values were not significantly different in cells transfected with the GFP plasmid compared to mock-transfected cells (not shown). E1A-expression levels rapidly declined over time (Supporting Fig. S4); loss of GFP expression was also observed, but at a slower rate. Interestingly, prostate cancer cells stably expressing E1A could not be generated, most likely because of the potent induction of cell death by constitutive E1A expression in these cells. To this end a recombinant Ad5 (ΔE1, ΔE3) expressing E1A12S under control of the CMV promoter was generated (AdE1A12S). Cells infected with the AdE1A12S virus expressed E1A at high and reproducible levels identical to that of Ad5 in all cell lines (data not shown). Combinations of AdE1A12S with mitoxantrone or docetaxel at four constant ratios resulted in strong synergistic cell killing in PC-3 and DU145 cells (Fig. 2A). In fact, the CI values were lower in >50% of data points for AdE1A12S (CI = 0.50–0.8) compared to the corresponding treatments with Ad5 and as low as those of the *dl*1520 mutant (Supporting Table S2). These data demonstrate that expression of the small E1A12S protein is sufficient to cause synergistic cell killing in combination with mitoxantrone and docetaxel.

**Table 1:** EC_50_ values for mitoxantrone and docetaxel in prostate cancer cells transfected with E1A12S or GFP expressing plasmids.

	EC_50_ Mitoxantrone (nM)		EC_50_ Docetaxel (nM)	
	E1A12S	GFP	E1A12S	GFP
**PC3**	510±50	2120±120	15±4	30±10
**DU145**	54±12	150±18	9±3	20±8
**22Rv1**	42±15	130±22	0.8±0.5	2.0±0.6

Data from one representative experiment treated with mitoxantrone for 3 days after transfection with pcDNA plasmids expressing the respective proteins, n = 3.

### Mutants expressing the small E1A12S proteins deleted in the p300-, p400- or pRb-binding regions are cytotoxic to prostate cancer

Having established strong synergistic effects with the E1A12S-expressing mutant, various E1A-deletions were incorporated, focusing on the regions that bind to p400 (AdE1A1102), p300/CBP (AdE1A1104) and pRb (AdE1A1108). The deletions were selected based on the observed sensitization with the corresponding replicating mutants (*dl*1102, *dl*1104, *dl*1108) and previous reports indicating that these E1A-regions are involved in apoptotic cell killing [21], [23], [28], [29]. As expected, the mutants had significantly lower cell killing potency than the replicating viruses with EC_50_ values 10–50 times higher than Ad5 in all three cell lines (p<0.001) (Table 2) while the E1A-deleted *dl*312 mutant had EC_50_ values >1×10^5^ ppc. AdE1A1102 and AdE1A1108 had higher potency than other mutants in DU145 and PC-3 cells. AdE1A1104 caused the least cell killing in all tested cell lines similar to findings with the replicating *dl*1104 mutant (Table 2, Fig. 1B). Replication of AdE1A1102, AdE1A1104 and AdE1A1108 was either below the limit of detection (<20 pfu/cell) or significantly reduced (p<0.001) compared to the corresponding replicating mutants up to 72 h after infection (Fig. 2B). In agreement with these data no significant increases in viral genome amplification over time were detected (Fig. 2C). In contrast, the corresponding replicating viruses showed time-dependent genome amplification to similar levels as Ad5 in DU145 and PC-3 except *dl*1104, that was slightly attenuated in PC-3 and 22Rv1 cells. We previously demonstrated that the oncolytic mutants AdΔCR2 and *dl*922–947 deleted in the CR2-region similar to *dl*1108, had only slightly attenuated replication and genome amplification in proliferating normal primary NHBE and PrEC cells when compared to wild type virus [16], [40]. Interestingly, replication of the *dl*1102 and *dl*1104 mutants was significantly (p<0.005) attenuated in normal NHBE cells compared to Ad5 (Fig. 2D). As expected all E1A12S-expressing mutants rapidly killed both NHBE and PrEC cells without detectable replication (data not shown). In conclusion, in the prostate cancer cells none of the AdE1A12S mutants replicated and consequently cell killing was caused solely by E1A expression.

**Table 2:** EC_50_ values (ppc) for the replication-defective AdE1A12S, AdE1A1102, AdE1A1104 and AdE1A1108 viral mutants and wild type virus (Ad5).

	Ad5	Ad12S	Ad1102	Ad1104	Ad1108
**PC3**	102±10	3072±660	1710±450	5090±690	2100±250
**DU145**	8±2	240±25	148±35	255±50	80±20
**22Rv1**	1.8±0.5	5.2±1.1	6.1±2.0	13.3±3.5	5.5±3

Data are averages ± SD, n = 3, p<0.001 for all mutants vs Ad5 (t-test). The non-replicating *dl*312 and AdGFP control viruses had EC_50_ values >1×10^5^ ppc.

### The AdE1A1104 virus does not sensitise prostate cancer cells to cytotoxic drugs

**Figure 3:**
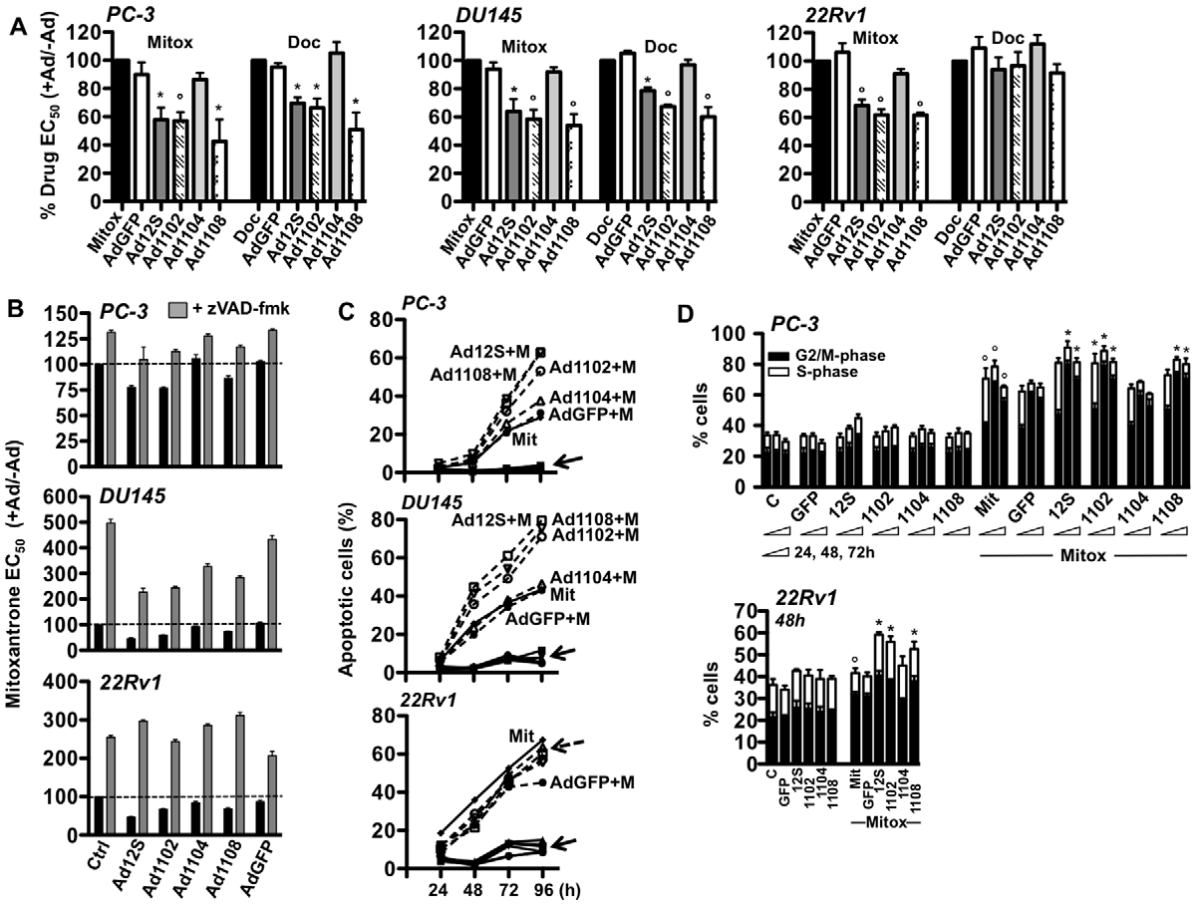
All replication-defective E1A12S mutants sensitise prostate cancer cells to mitoxantrone and docetaxel except the AdE1A1104 virus. A) Drug dose responses in each cell line were evaluated after infection with AdE1A12S, AdE1A1102, AdE1A1104, and AdE1A1108 mutants with AdGFP as negative control to determine changes in drug EC_50_ values. All cell lines were infected at doses killing <10% of cells alone; PC-3 cells at 100 ppc (left panel), DU145 cells at 10 ppc (mid panel) and 22Rv1 cells at 2.5 ppc (right panel). Data represent averages ±SD, n = 4–5 independent experiments analysed by t-test comparing EC_50_ values for each combination to that of drug alone, expressed as percentages, *p<0.05 and °p<0.01. B) EC_50_ values for mitoxantrone were determined with and without simultaneous infection with viral mutants at 2.5, 10 and 100 ppc for 22Rv1, DU145 and PC-3 respectively, and with (grey bar) and without (black bar) the addition of the pan-caspase inhibitor zVAD-fmk at 25 µM. EC_50_ values are expressed as percentages of mitoxantrone alone (Ctrl), averages ± SD, n = 3. C) Flow cytometry of cells infected with the AdE1A12S mutants or treated with mitoxantrone (50 nM) alone and in combination and analysed for tetramethylrhodamine uptake (TMRE) as an indicator of mitochondrial depolarisation and apoptosis induction. AdE1A12S, AdE1A1102, AdE1A1104, AdE1A1108, and AdGFP alone (solid arrow; all cell lines) and AdE1A12S, AdE1A1102, AdE1A1104 and AdE1A1108 in combination with mitoxantrone (dashed arrow; 22Rv1 cells). Data expressed as % apoptotic cells; percentages of cells that showed mitochondrial depolarisation, averages ± SD, n = 3. D) Cells infected with each mutant at 100 (PC-3) or 2.5 ppc (22Rv), mock infected and treated with or without mitoxantrone at 50 nM. Changes in cell cycle were analysed by flow cytometry at 24, 48 and 72 h after infection and drug treatment in PC-3 cells or after 48 h for 22Rv1 cells, one representative study (n = 3), *p<0.05 comparing G2/M-phase in combination treated vs mitoxantrone alone, °p<0.05 G2/M-phase for mitoxantrone vs mock treated.

Next, the 22Rv1, DU145 and PC-3 cells were infected with the non-replicating viral mutants at doses that caused <10% cell killing alone, at 2.5, 10 and 100 ppc respectively, and treated with increasing doses of mitoxantrone or docetaxel (Fig. 3A). In PC-3 and DU145 cells all mutants, except AdE1A1104, were as potent as the intact AdE1A12S virus and significantly decreased the drug EC_50_ values by 40–55% for mitoxantrone and 30–50% for docetaxel. Neither AdE1A1104 nor AdGFP sensitized the cells to any drug. In the more sensitive 22Rv1 cells the EC_50_ values were significantly decreased for mitoxantrone with all mutants except AdE1A1104 and AdGFP, but not for docetaxel. However, a trend towards sensitisation with AdE1A12S, AdE1A1102 and AdE1A1108 was detected at higher doses (10 ppc; not shown). The differences in efficacy were not caused by differences in virus integrity since the vp/pfu ratios were 19–40 for all mutants (Supporting Table S1) with the highest activity for AdE1A1104 (19 vp/pfu). In addition, the same trends were observed at both lower and higher doses of all mutant viruses in PC-3 and DU145 (data not shown).

### E1A-induced sensitisation to mitoxantrone is dependent on apoptotic cell death

Both mitoxantrone and docetaxel ultimately kill cancer cells through activation of apoptotic mechanisms resulting from DNA damage [41], [42]. Expression of E1A alone in the absence of E1B or other viral proteins has been reported to potently induce apoptosis in various cell types (*e.g.* [21], [25], [26]). To determine if caspase-dependent apoptosis was involved in the E1A-mediated sensitisation in prostate cancer cells, cells were infected with AdE1A12S mutants and treated with mitoxantrone under synergistic conditions with and without the addition of the pan-caspase inhibitor v-ZAD-fmk (Fig. 3B). Mitoxantrone-induced cell killing was greatly reduced in all cells treated with the inhibitor. In combination-treated cells the sensitization was completely blocked by the caspase inhibitor. Despite the lack of sensitization to mitoxantrone with the AdE1A1104 and AdGFP mutants, cell viability increased with the inhibitor by preventing drug-induced apoptosis (Fig. 3B). To further investigate the molecular mechanisms leading to caspase activation, early changes in mitochondrial membrane depolarisation were determined by tetramethylrhodamine uptake (TMRE). Low doses of each AdE1A12S mutant resulting in <10% of cells with mitochondrial depolarisation up to 96 h after infection were combined with a dose of mitoxantrone that induced potent depolarisation in all tested cell lines (Fig. 3C). In PC-3 and DU145 cells, AdE1A12S, AdE1A1102 and AdE1A1108 increased the percentages of depolarised apoptotic cells in combination with mitoxantrone by up to 30% after 96 h compared to drug alone. In contrast, the AdE1A1104 mutant did not further increase the mitoxantrone-induced apoptosis and had similar effects to the control AdGFP virus (Fig. 3C). Interestingly, no combination increased mitochondrial depolarization in 22Rv1 cells compared to treatment with drug alone under these conditions. We conclude that although the small E1A12S mutants promoted potent caspase-dependent apoptotic death in all three cell lines the early apoptotic events differed in response to the combination treatments with mitoxantrone; the mitochondrial pathway appeared to be involved in the sensitization in PC-3 and DU145 cells but not in 22Rv1 cells. In addition, despite increased viability in all combination treated cells infected with AdE1A1104 in the presence of caspase inhibitor, the mitochondrial pathway appeared not to be activated by this mutant.

### AdE1A12S, AdE1A1102 and AdE1A1108 but not AdE1A1104 promote mitoxantrone-dependent G2/M-induction

To further investigate the differences in sensitization, we determined the cell cycle distribution during synergistic conditions. The viral mutants caused minor (not significant) increases in S- or G2/M- phases in PC-3 and 22Rv1 cells (Fig. 3D). Mitoxantrone increased the G2/M-population in all cell lines as previously reported for this topoisomerase II inhibitor [41]. In PC-3 cells, the viral mutants that caused sensitization (AdE1A12S, AdE1A1102, AdE1A1108) further increased the drug-induced G2/M-population from 24 to 72 h in combination with mitoxantrone but not AdE1A1104 (Fig. 3D). The same changes were observed in 22Rv1 cells with the greatest effects 48h after treatment initiation (Fig. 3D). Cell cycle profiles with AdE1A1104 or AdGFP in combination with mitoxantrone were similar to that of mitoxantrone alone in both cell lines. The increases in G2/M were greatest in the PC-3 cells and likely reflect the more potent sensitization of these cells. In DU145 cells, combinations of the AdE1A12S mutants with mitoxantrone resulted in a high fraction of aneuploidy and greatly increased subG1- and G2/M- phases already after 24 h making the distinction between phases difficult (data not shown). However, the increased aneuploidy and G2/M populations were more evident with AdE1A12S, AdE1A1102 and AdE1A1108 than with AdE1A1104. In agreement with the cell cycle data, mitoxantrone induced cyclin A and B levels as would be expected for cells in the G2/M-phase (Supporting Fig. S5). While the drug-induced increases in cyclin levels were sustained with all E1A12S mutants, no further changes could be detected in the combination treated cells. Overall no effects on drug-induced cell cycle alterations or apoptotic cell killing could be detected in AdE1A1104 infected cells.

### In combination with docetaxel both dl1102 and dl1104 inhibit PC-3 tumor xenograft growth and prolong time to tumor progression in athymic mice

**Figure 4:**
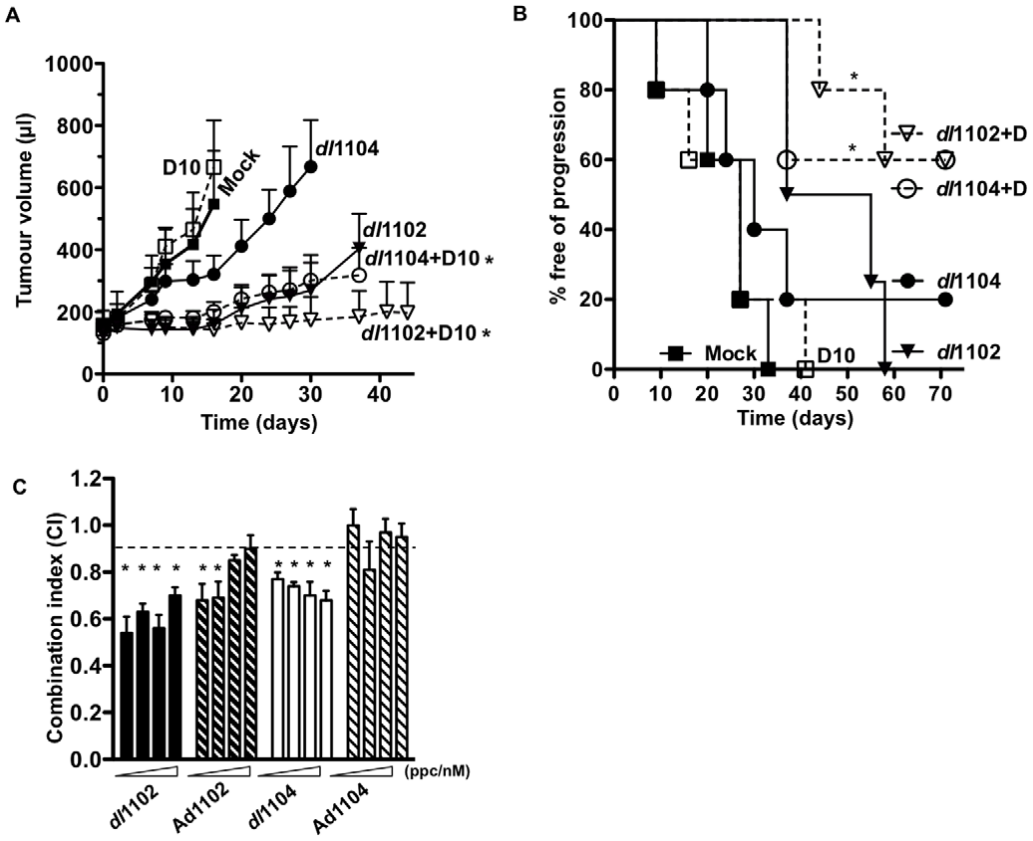
The dl1102 mutant prolongs time to progression in combination with docetaxel in PC-3 xenografts *in vivo.* A) Animals with PC-3 subcutaneous tumor xenografts were treated with the *dl*1102 (filled triangle) or *dl*1104 (filled circle) mutants or mock treated with *dl*312 (filled square) at 1×10^9^ vp (i.t. injections on day 1, 3, and 5) with and without docetaxel at 10 mg/kg (D10; i.p. administration on day 2 and 8, open squares), and tumor growth was monitored. *p<0.05, treatments compared with mock and single-agent treatments (one-way ANOVA), p<0.05 for *dl*1102 alone compared to mock, n = 6. B). In a second study animals with PC-3 subcutaneous tumor xenografts were treated as above with the indicated suboptimal doses of mutants at 1×10^9^ vp and docetaxel at 10 mg/kg (D10) or the respective combinations. Median time to tumor progression (tumor volume >500 µl) was determined by Kaplan-Meier survival analysis (8–10 animals per group). *p<0.05, combination-treated compared with docetaxel. C) PC-3 cells infected with the indicated mutants and treated with docetaxel at four constant ratios; 0.5, 2.5, 12.5 and 62.5 ppc/nM drug (indicated by the wedges). CI values were calculated from isobolograms and CI≤0.9 were considered synergistic, averages ±SEM, n = 3, *p<0.05 vs the theoretical additive values (0.9<CI<1.1) represented by the dashed line.

To explore our findings *in vivo,* PC-3 cells were inoculated subcutaneously in athymic mice as previously described [16]. As expected, the replication-defective AdE1A12S, AdE1A1102 and AdE1A1104 mutants did not have significant anti-tumor efficacy in this model due to the lack of viral replication and spread, neither alone nor in combination with docetaxel (not shown). In contrast to docetaxel, mitoxantrone was too toxic for evaluation in the PC-3 *in vivo* model. Differently from the replication-defective mutants, the replicating *dl*1102 and *dl*1104 potently inhibited tumor growth in combination with docetaxel at low doses (Fig. 4A; p<0.05 compared to each single agent). However, only *dl*1102 had significant efficacy (p<0.05 compared to mock treated) when administered alone. In a second study, median time to progression was determined to be 40, 30 and 28 days for *dl*1102, *dl*1104 and docetaxel respectively. In contrast, more than 50% of the combination-treated animals had still not progressed at the end of the study, 70 days after treatment (Fig. 4B). The enhanced anti-tumor efficacy observed *in vivo* was verified in cultured PC-3 cells treated with viral mutants and docetaxel. Combination-treatments with docetaxel caused similar synergistic effects as observed with mitoxantrone. The most potent synergistic cell killing was achieved when docetaxel was combined with *dl*1102 (p<0.05) and to a lesser degree with *dl*1104 (Fig. 4C). The replication-defective AdE1A1102 was less potent than the replicating *dl*1102 with synergy in two data points (p<0.05) while AdE1A1104 did not cause significant synergy.

### The replicating dl1102 and dl1104 mutants do not enhance cell killing in combination with cytotoxic drugs in primary human prostate (PrEC) and bronchial (NHBE) epithelial cells

**Figure 5:**
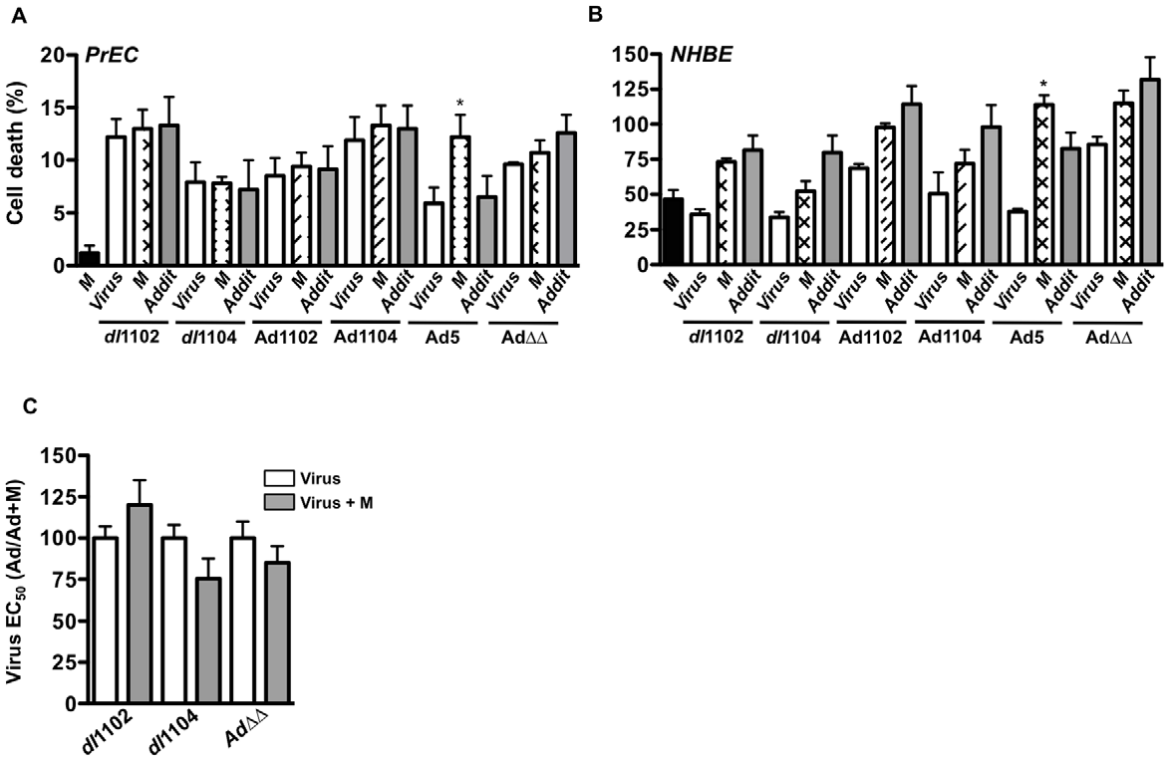
Primary human prostate (PrEC) and bronchial (NHBE) epithelial cells are not sensitive to combinations of viral mutants and cytotoxic drugs. A–B) Normal human prostate (PrEC; A) and bronchial (NHBE; B) epithelial cells were infected with the replicating *dl*1102, *dl*1104, Ad5wt, AdΔΔ and the non-replicating Ad1102 and Ad1104 at 10 ppc alone (white bars) and in combination with mitoxantrone (M) at 200 nM (crossed and striped bars). Cell viability was determined by the MTS assay 72 h later. The theoretical additive (Addit) values are indicated by grey bars, *p<0.05 compared to expected additive cell killing, n = 2. C) PrEC cells were infected at increasing doses with the *dl*1102, *dl*1104 and the oncolytic mutant AdΔΔ with and without the addition of 300 nM mitoxantrone (M). Data are presented as the percentages of the viral EC_50_ values in combination with mitoxantrone compared to virus alone, n = 3. Mitoxantrone alone caused 2–8% cell death and was corrected for in the calculations.

In contrast to the cancer cell lines, no significant enhancement of cell killing was observed in normal primary PrEC or NHBE cells with any mutants in combination with mitoxantrone (Fig. 5A–B) or docetaxel (not shown). In agreement with our previous findings, Ad5 wild type virus sensitized both PrEC and NHBE cells to the drugs while no significant sensitization was observed with the replication-selective oncolytic AdΔΔ mutant as previously reported [16], [40]. A trend towards increased cell killing was noted for *dl*1104, but not with other mutants, in combination with higher doses of mitoxantrone (Fig. 5C). Taken together, these findings suggest that both *dl*1102 and *dl*1104 are safe with low toxicity to normal tissue and have potential for future developments of oncolytic mutants targeting prostate cancer. However, efficacy for the *dl*1104 mutant administered alone was poor and significantly less than with *dl*1102.

## Discussion

The findings presented here demonstrate potent E1A-mediated chemosensitization in three prostate cancer cell lines, with and without functional AR and p53 pathways. We show that expression of the small viral E1A12S protein from a replication-defective virus or plasmid is sufficient to synergistically enhance cell killing in combination with mitoxantrone or docetaxel. The contribution of specific E1A-domains in the enhancement of drug-induced apoptosis was determined in the absence of the E1ACR3 transcriptional activation domain. We demonstrate that a region within E1ACR1 (amino acids 48–60) is essential for drug-sensitization, while both E1ACR2 and a region proximal to CR1 (amino acids 26–35) are redundant in our prostate cancer models. We also demonstrate that a panel of E1A13S-expressing, fully replicating deletion-mutants, previously established as defective in binding to pRb, p400/TRRAP, PCAF, p21 or p300/CBP [24], [30], [31], [35], [36], [37], sensitized the cells to cytotoxic drugs to different degrees dependent on the specific cell line. Importantly, the replicating *dl*1108 and *dl*922–947 (ΔE1ACR2), and the *dl*1102 (amino acids 26–35 deleted) mutants consistently killed all tested prostate cancer cells and sensitized the highly treatment-resistant PC-3 cells to both mitoxantrone and docetaxel to a greater degree than wild-type virus. In contrast, *dl*1104 and *dl*1101 (amino acids 26–35 and 4–24 deleted, respectively) had lower cell killing potency and were only slightly better than the attenuated *dl*1520 virus. Interestingly, while the corresponding non-replicating AdE1A12S, AdE1A1102 and AdE1A1108 also potently enhanced mitoxantrone- and docetaxel- induced cell killing, the AdE1A1104 mutant did not sensitize any cells. Binding of cellular factors to specific amino acid motifs in E1A have previously been elegantly demonstrated by numerous researchers, therefore, we did not perform additional binding assays in this study (reviewed in [11], [30]). Taken together with previous reports, our findings indicate that binding of p300/CBP to the E1A12SCR1 domain is the most likely factor to play a role in sensitization with AdE1A1102, AdE1A1108 and AdE1A12S. Interestingly, when the CR1 domain was deleted in the large E1A13S protein as in *dl*1104, sensitization was observed, albeit at a lower level. A possible reason might be the recently discovered additional binding site for the p300/CBP complex in the E1ACR3-domain (present in E1A13S) [32]. Binding of p300/CBP to E1A13SCR3 mainly contributes to transcriptional activation, while binding to the E1A12SCR1 region suggests transcriptional repression [32], [43].

The cellular mechanisms involved in E1A-dependent drug-sensitization are elusive, mostly because numerous cellular proteins can bind to overlapping regions in E1A and have related functions. Several histone acetyl transferases (HATs) bind to the N-terminal and CR1 domains of E1A including p400, p300/CBP, PCAF, TRRAP to name a few. HATs are major regulators of cellular functions and E1A-binding to HATs interferes with normal cell homeostasis, for example, selective transcriptional activation/repression by E1A-binding to p400 and a more general transcriptional repression by binding to p300/CBP. The E1A p300/CBP complex acts as a scaffold to TFIID preventing transcription factor binding to TATA domains [28], [44]. Lack of p300/CBP binding to E1A12S (AdE1A1104) attenuates protein degradation and increases the levels of c-myc and E2F, while lack of p400 binding (AdE1A1102) would result in increased protein degradation through higher expression of HDM2 and ubiquitination [45]. Overall, binding of either p400 or p300/CBP to E1A12S inhibits p21-mediated cell cycle arrest and promotes cell cycling in the presence of DNA-damage ultimately resulting in apoptotic death [11], [28], [30], [31]. However, none of the viral mutants significantly affected p21 levels under our conditions and did not prevent mitoxantrone-induced increases of p21, while E1A12S and AdE1A1102 but not AdE1A1104 promoted degradation of the AR in 22Rv cells within 24 h (data not shown). The AR is partly stimulated by p300 acetylation and is attenuated by histone deacetylases (HDACs) and MDM2-mediated degradation [46]. Although, we did not explore the exact role of E1A-p300/CBP binding in drug sensitization we speculate that cellular protein-degradation, including growth-stimulating factors such as the AR, is promoted by opposing cellular factors when the p300/CBP enzyme activity is squelched by E1A and thereby contributing to cell death.

The E1A-p300/CBP complex also represses p53-dependent transcription, in turn preventing cell cycle arrest to support high levels of viral replication [45], [47]. A trend towards lower levels of replication for *dl*1104 was noted in the p53-positive 22Rv cells. The AdE1A1104 mutant was the only virus that did not further increase the mitoxantrone-induced G2/M cell population or aneuploidy. In contrast, mutants lacking the pRb- or p400- binding domains were as effective as the intact AdE1A12S in supporting the accumulation of cells in G2/M in both PC-3 and 22Rv1 cells. The already deregulated cell cycle in these cells is apparently sufficient to compensate for the absence of pRb-E1A and p400/p21-E1A complexes. E1ACR1-deleted mutants have been reported to undergo more rapid proteasomal degradation than wild-type E1A [48]. We noted a slightly lower level of immunoreactive E1A expressed from the AdE1A1104 mutant and to rule out a dose-dependent effect on sensitization we infected cells with increasing doses. However, sensitization to drugs was still not observed (data not shown). These results suggest that infection with all mutants under our conditions resulted in E1A-expression that reached the critical threshold required for cellular effects and consequently, the attenuated potency of E1A1104 mutants is caused by the absence of binding to p300/CBP or other cellular factors.

Additional factors that interfere with E1A-induced chemosensitization are the many genetic alterations present in cancer cell lines. For example, binding of p300/CBP was previously reported not to be essential for apoptosis-induction while p400-binding enhanced the sensitization to adriamycin in primary mouse embryo fibroblasts (MEF) [28]. Similar findings were also observed in primary transformed retinal cells [49]. Induction of apoptosis and sensitization was reported to be dependent on p300 or p400 stabilization of p53 through an ARF-mediated mechanism and by induction of E2F or c-Myc [28], [49]. However, our findings presented here clearly demonstrate that E1ACR1 (p300-binding; Δ48–60) was essential for enhancement of drug-induced apoptosis but not the p400-binding region (Δ26–35) and E1ACR2 (pRb-binding; Δ122–129) in our prostate cancer models. A major difference between our study and previous reports is the use of prostate cancer cell lines and the cytotoxic drugs mitoxantrone and docetaxel, explored here for the first time with these mutants. We speculate that the deregulated signalling pathways in prostate cancer cells, including aberrant control of cell cycle progression and death, compensate for many of the E1A-functions that are required for sensitization in normal or transformed cells. Importantly, we demonstrate that non-replicating E1A12S and fully replicating mutants with the p300 or p400 binding domains ablated could not sensitize normal cells, neither PrEC nor NHBE, to the cytotoxic drugs. Furthermore, *dl*1102 and *dl*1104 replication was significantly attenuated in the NHBE cells. Together with our previous reports demonstrating that replicating viruses deleted in the E1ACR2 domain (AdΔΔ and *dl*922–947) do not sensitize NHBE cells to cytotoxic drugs, these findings are important for future engineering of oncolytic viruses without toxicity to normal tissue [16], [40]. Previously, the E1A N-terminal and CR1 domains were reported as essential for apoptosis-induction, while the role of E1ACR2 was not clearly determined [29], [50]. In this report and in our previous studies we showed that the E1ACR2 region is redundant for sensitization in prostate cancer cells both in the replication-defective AdE1A1108 and replication-selective *dl*922–947 and AdΔΔ mutants [16], [19]. Recent findings suggest that CR1 and CR2 domains might cooperate in binding to cellular factors [11], [32], [51], further supporting our observations that the CR1 domain is important both for viral potency and for interaction with cellular factors.

In combination with the cytotoxic drugs all non-replicating mutants, except AdE1A1104, induced caspase-dependent apoptosis in all three cell lines although, mitochondrial membrane depolarisation was only increased in DU145 and PC-3 cells and appeared to be dependent on the presence of the p300/CBP binding domain. The lack of further increases in mitochondrial depolarisation in 22Rv1 cells indicate that apoptosis is induced through direct caspase activation in these cells for example, by E1A-mediated caspase 8 and 3 activation through E1A-binding to the caspase 8 inhibitor cFLIP [52]. Activation of the seemingly different pathways in the three cell lines is likely the consequences of specific genetic alterations in each cell line [5], [8]. The most obvious differences are the functional p53 pathway and AR signalling in 22Rv1 cells but not in PC-3 and DU145 cells. It is possible that the presence of p53 renders these cells more sensitive to both E1A- and drug-induced cytotoxicity, reflected in the significantly lower EC_50_ values for both sets of compounds in 22Rv cells. Furthermore, 22Rv1 cells are more infectible than PC-3 cells [19]. Extensive in depth studies would be required to delineate the signalling cascades that cause the observed differences in each cell line and with each mutant, even though overall enhancement of cell killing is the final result in all three cell lines.

We have for the first time demonstrated that the small E1A12S protein alone can sensitize prostate cancer cells to mitoxantrone and docetaxel and that a mutant without the p400/p21-binding domain (AdE1A12S1102) caused similar potent sensitization and enhanced apoptosis. The corresponding replicating *dl*1102 mutant had higher potency than wild-type virus in synergy assays with both drugs. Interestingly, the *dl*1102 mutant alone, but not *dl*1104, significantly reduced tumor growth in the PC-3 xenograft model *in vivo*. Both *dl*1102 and *dl*1104 were highly efficacious in combination with docetaxel and significantly prolonged time to progression. Even though the deregulated cell cycle control in PC-3 cells is sufficient to support replication of all tested mutants including the ΔCR2 mutants, viral efficacy was attenuated for the *dl*1104 virus when given alone. Overall, efficacy in our experimental models was significantly higher with the replicating *dl*1102 mutant compared to *dl*1104, both when given alone and in combination with mitoxantrone and docetaxel. In addition, replication of this mutant was significantly attenuated in the normal NHBE cells and no sensitization to drugs was observed in normal cells.

To date, the choice of E1A-deletions has been defined by the genetic complementation of deregulated cellular pathways such as the potent AdΔCR2 viruses. However, improvements in future therapies for prostate cancer will likely include multimodal strategies and we suggest that to optimise efficacy, the intrinsic sensitizing properties of E1A and deletion of small protein-binding domains such as the p400/p21-binding region, should be investigated in combination with the highly potent and selective AdΔCR2 mutants.

## Materials and Methods

### Cancer cell lines

The human prostate carcinoma cell lines PC-3 (ECACC, UK), DU145 and 22Rv1 (ATCC, USA), the murine prostate cell lines TRAMP-C1 (mouse transgenic Probasin-TAg prostate cancer; ATCC) and RM1 (ras/myc-transformed; kind gift from Prof T.C. Thompson, Baylor College of Medicine, Houston, TX [53]) were grown in Dulbecco's Modified Eagle Medium (D-MEM) supplemented with 10% foetal calf serum (FCS). All cell lines were authenticated by STR-profiling (Cancer Research UK and LGC Standards, UK) and verified to be identical to the profiles reported by ATCC and the original vials at the end of the studies. The primary normal human prostate (PrEC) and bronchial (NHBE) epithelial cells were cultured according to the manufacturer's instructions (Lonza).

### Adenoviruses

All replicating E1A-deletion mutants were serotype 5 (Ad5), based on the *dl*309 backbone (E3B-deleted) with the following E1A amino acid deletions: *dl*1101 (ΔE1A4–25), *dl*1102 (ΔE1A26–35), *dl*1104 (ΔE1A48–60 in CR1), *dl*1107 (ΔE1A111–123), *dl*1108 (ΔE1A124–127 in CR2) and *dl*922–947 (ΔE1A122–129 in CR2). The *dl*1101–1108 series of mutants were kind gifts from Prof. S.T. Bayley and Prof. J.S. Mymryk [35], [36], [37]. The selectively replicating *dl*1520 mutant (ΔE1B55K, ΔE3B), Ad5 (wild type), non-replicating AdGFP (ΔE1) and *dl*312 (ΔE1A, ΔE3B) were used as controls. All viruses had a viral particle to infectious unit ratio of 10–40 vp/pfu.

### Cell killing assay and synergistic interactions

Dose response curves to viral mutants, mitoxantrone (Onkotrone; Baxter) and docetaxel (Taxotere; Fluka) were generated by serial dilutions to determine the concentrations killing 50% of cells (EC_50_). Cell viability and cell killing efficacy were analysed 3–6 days after treatment using the MTS-assay (Promega). Synergistic interactions were determined at four constant dilution ratios of viruses and drugs at 0.5, 2.5, 12.5 and 62.5 viral particles per cell (ppc)/nM drug and isobolograms were generated from individual EC_50_ values followed by determination of combination index (CI) as previously described [16], [19], [39]. Each data point was determined from triplicate samples, and repeated 3–5 times. Synergy was defined as a greater effect on cell death than the theoretical additive values; CI≤0.9 = synergy (S), CI≥1.1 = antagonism (A) and 0.9<CI<1.1 additive (Add) effect [54]. In sensitisation studies the cells were treated with serial dilutions of drugs and fixed doses of viral mutants at 2.5, 10 and 100 ppc in the 22Rv1, DU145 and PC-3 cells respectively, or with serial dilutions of virus and fixed doses of drugs at 10 or 50 nM for mitoxantrone and 0.1 or 1.0 nM for docetaxel. Data are presented as percentages of the EC_50_-values for drug or virus alone after correction for cell death induced by the corresponding control (virus or drug alone; <15%). The pan-caspase inhibitor zVAD-fmk (Calbiochem/Merck, UK) was added at 25 µM to inhibit cell killing.

### Generation of E1A-expressing vectors and replication-defective E1A12S-mutants

Total RNA was isolated from A549 cells infected with Ad5 at 100 ppc for 24 h (Trizol Reagent; Invitrogen), cDNA was synthesized with TaqMan Reverse Transcription Reagent and oligo(dT) primers (Applied Biosystems), amplified with E1A primers and cloned into a pCR2.1-TOPO vector (Invitrogen). E1A12S inserts were verified by sequence analysis, cloned into pShuttle-CMV vectors (Stratagene, TX, USA) and were either used to transfect prostate cancer cells directly with the JetPEI-RGD reagent (PolyPlus) or were further linearised and recombined with a pAdEasy-1 plasmid (ΔE1, ΔE3; Stratagene) into the E1A site according to the manufacturer's instructions. The AdE1A1102, AdE1A1104 and AdE1A1108 mutants were generated by gene splicing by overlapping extension PCR (SOEing PCR) using E1A12S as the template. The PCR fragments were cloned into a pCR2.1-TOPO vector, sequenced and further cloned into the pShuttle-CMV vector and recombined with the pAdEasy-1 plasmid. All recombinant viral DNA was isolated, linearized and transfected into HEK293 cells. The resulting viral mutants were analyzed, characterized and sequenced to verify the specific inserts and deletions as previously described [16].

### Replication assay

Human prostate cancer cells were seeded at 2×10^5^ cells/well in 6-well plates and 24 h later infected with viruses at 10–100 ppc. Cells and media were collected at 24–72 h post-infection, freeze-thawed and analyzed by the tissue culture inhibitory dose at 50% (TCID_50_) using JH293 cells, as previously described [55]. Each sample was determined in triplicate and data from three independent studies were averaged and expressed as pfu/cell ± SD.

### Quantitative PCR (qPCR)

Cells were infected with viral mutants at 10–100 ppc and DNA extracted 3, 24, 48 and 72 h after infection using the DNA blood extraction kit (Promega). Viral genomes were quantified in 10 ng of sample DNA with the following primers: hexon-forward; 5′-GGACAGGCCTACCCTGCTAAC-3′, hexon-reverse; 5′- TGCTGTCAACTGCGGTCTTG-3′. Power SYBR Green Master Mix was added and qPCR performed (7500 Real Time PCR System; Applied Biosystems). Results were expressed as the ratio of viral genome copies at each time point relative to that at 3 h after infection as previously described [16].

### Reverse transcription (RT)-qPCR

The 22Rv1, DU145 and PC-3 cells were infected with viral E1A-mutants at 2.5, 10 and 100 ppc respectively and/or treated with mitoxantrone at 50 nM for 24 h followed by RNA extraction (Trizol Reagent). First-strand cDNA was synthesized from 1 µg of total RNA using MMLV-Reverse transcriptase and random hexamer primers for E1A and 18S RNA as previously described [56]. The qPCR was performed as described above and results were expressed as the ratio of E1A cDNA to cellular 18S cDNA (g/g×10^3^) in each sample, n = 3.

### Flow cytometry analysis

Cells were infected with AdE1A-mutant viruses at 10–100 ppc and/or treated with mitoxantrone at 50 nM or docetaxel at 1 nM and harvested 24–96 h later. For cell cycle analysis, cells were fixed (70% ethanol, 5 µg RNase A) and analysed on a FACSCalibur instrument (Becton Dickinson) after addition of 10 µg propidium iodine (PI). Changes in mitochondrial membrane potential (Δψ) were determined by staining with tetramethylrhodamine ethyl ester perchlorate (TMRE; Molecular Probes/Invitrogen) at 60 ng/ml in PBS containing 4–6-diamidino-2-phenylindole (DAPI) at 1 µg/ml and analyzed on an LSRI (Becton Dickinson), previously described [56].

### Immunoblot analysis

Cells were treated with viruses and drugs as described above, harvested and lysed 24–72 h post-infection (25 mM Tris-HCl, 150 nM NaCl, 1 mM EDTA, 1 mM DTT, 1 mM NaF, 1% NP-40 (v/v) 1% sodium deoxycholate, 0.1% SDS containing a protease inhibitor cocktail; Roche). Total proteins, 10–20 µg, were separated on SDS-polyacrylamide gels under reducing conditions, transferred to polyvinylidene fluoride membranes (PVDF; Invitrogen) and detected with the following antibodies: cyclins A, B and D at 1∶200 (Santa Cruz Biotechnology), rabbit anti-Ad2/5 E1A at 1∶200 (SC-430), rabbit anti-hexon at 1∶2000 (AutogenBioclear), mouse anti-ß-tubulin at 1∶20000 (Sigma-Aldrich) and goat anti-actin at 1∶1000 (SC-1615). Detection was by horseradish peroxidase-conjugated secondary (Dako) antibody as appropriate and chemiluminescence reagent (Amersham/Pharmacia) followed by autoradiography (BioMax film; Kodak).

### *In vivo* tumor growth

Five to six week old male C57Bl/6 athymic (ICRF *nu/nu*; CR UK) mice were maintained in individually ventilated cages (IVC) equipped with bedding and stress reducing modules. Animals had free access to food and water at all times. Inoculation of tumor cells and all injections were performed on anesthetized animals using an isoflurane vaporizer delivering 2–3% isoflurane, oxygen and nitrous oxide in air. Tumors were grown in one flank by subcutaneous implantation of 1×10^7^ PC-3 cells as previously described [16]. When tumors were 100±20 µl animals were randomised into treatment groups of 7–10 animals/group. Dose responses to viral mutants and docetaxel were determined by intratumoral administration (i.t.) of 1×10^8^–1×10^9^ vp/injection/100 µl in PBS three times at 48 h intervals and docetaxel at 10.0 mg/kg in 100–200 µl PBS intraperitoneally (i.p.) two times from days 2–10 after the first virus injection. Low doses of viruses and docetaxel were selected to enable detection of additive/synergistic effects on tumor growth inhibition. Tumor volumes were estimated twice weekly: volume = (length×width^2^×π)/6. Tumor growth and progression were monitored for 3 months or until tumors reached ≤1.44 cm^2^, at which point animals were terminated in accordance with the UK Home Office Regulations using isoflurane. Differences in tumor growth between treatment groups were analysed by one-way Anova and p-values <0.05 were considered significant. Time to progression (tumor volume ≥500 µl) was determined according to the Kaplan-Meier method (log rank test for statistical significance).

### Ethics statement

All animal studies were carried out in strict accordance with the UK Home Office Guidelines for Animals (Scientific Procedures) and the UKCCCR Guidelines for the Welfare of Animals in Experimental Neoplasia. All protocols were approved by the Committee on the Ethics of Animal Experiments of Queen Marys University London under the Home Office project license PPL 70/6393.

## Supporting Information

Figure S1 **Cell killing efficacy of replication-selective E1A-deletion mutants in the murine prostate cancer cell lines, TRAMP-C1 and RM1.** Viral EC_50_ values were determined from dose-response assays and presented as averages ± SD, n = 3. Significantly (1-way Anova) different values compared to Ad5 are indicated; (*) p<0.05 and (**) p<0.01. The dashed line represent the corresponding value for Ad5 in the least sensitive human prostate cancer cell line PC-3.
(TIFF)

Figure S2 **The PC-3 cells are insensitive to mitoxantrone and docetaxel.** A) Sensitivity to the cytotoxic drugs mitoxantrone and docetaxel in the human prostate cancer cell lines DU145, PC-3 and 22Rv1. B) Sensitivity to mitoxantrone and docetaxel in the murine prostate cancer cells TRAMPC and RM1. The dotted lines represent the corresponding EC_50_ values for the drug insensitive and sensitive PC-3 and 22Rv1 cells respectively. A–B) Data presented as EC_50_ values (6 days after addition) in each cell line, averages ± SD, n = 3.
(TIFF)

Figure S3 **Potent cell killing of the murine prostate cancer cells TRAMPC infected with replicating E1A-deletion mutants in combination with mitoxantrone.** A) Sensitization of the TRAMPC cells to mitoxantrone by fixed doses of each virus at EC_10_ and EC_25_. Data presented as percentages of mitoxantrone EC_50_ values in each cell line, averages ± SD, n = 3. Statistical analysis by 1-way Anova, *p<0.05 for drug EC_50_ values that were significantly lower than the corresponding Ad5 values. The *dl*312 (ΔE1A) non-replicating virus served as negative control. B) Graphic representation of combination indexes (CI) generated from synergy studies with mitoxantrone in combination with each replicating viral mutant at two constant ratios 0.5 and 2.5 viral particles per cell (ppc)/nM drug. Synergistic interactions are represented by CI≤0.9, antagonism by CI≥1.1 and additive effects by 0.9<CI<1.1, averages ± SEM, n = 3–5, *p<0.05 by t-test compared to the theoretical additive values.
(TIFF)

Figure S4 **E1A-levels decrease over time after transfection with the E1A12S expressing plasmid.** Expression levels of E1A in 22Rv1 cells transfected with pcDNA-12S, cells were harvested 24 h–6 d after transfection and E1A identified by immunoblotting. Ad5 infected cells were used as a control for E1A expression that was maximal after 48 h. One representative experiment (n = 3).
(TIFF)

Figure S5 **Mitoxantrone-induced G2/M phase is paralleled by increases in cyclins A and B in PC-3 cells.** Immunoblot illustrating changes in expression levels of cyclin A, B and D in cells infected with 100 ppc of each mutant and treated with 50 nM Mitoxantrone for 48 h. Total protein 20 µg/lane was loaded and blotted with the respective antibody as described in Material and Methods. No significant differences were detected between the mutants. Representative blot (n = 4).
(TIFF)

Table S1 **Ratio of viral particle (vp) to replicating virus (pfu).**
(DOC)

Table S2 **Combination index (CI) for Ad5, AdE1A-12S or dl1520 in combination with mitoxantrone or docetaxel in the human prostate cancer cells PC3 and DU145.**
(DOC)
