# Supplementary figures and images for: Adenovirus-Mediated Sensitization to the Cytotoxic Drugs Docetaxel and Mitoxantrone Is Dependent on Regulatory Domains in the E1ACR1 Gene-Region
Source: PLoS One. 2012 Oct 3;7(10):e46617. doi: 10.1371/journal.pone.0046617 (PMC3463540; doi:10.1371/journal.pone.0046617)

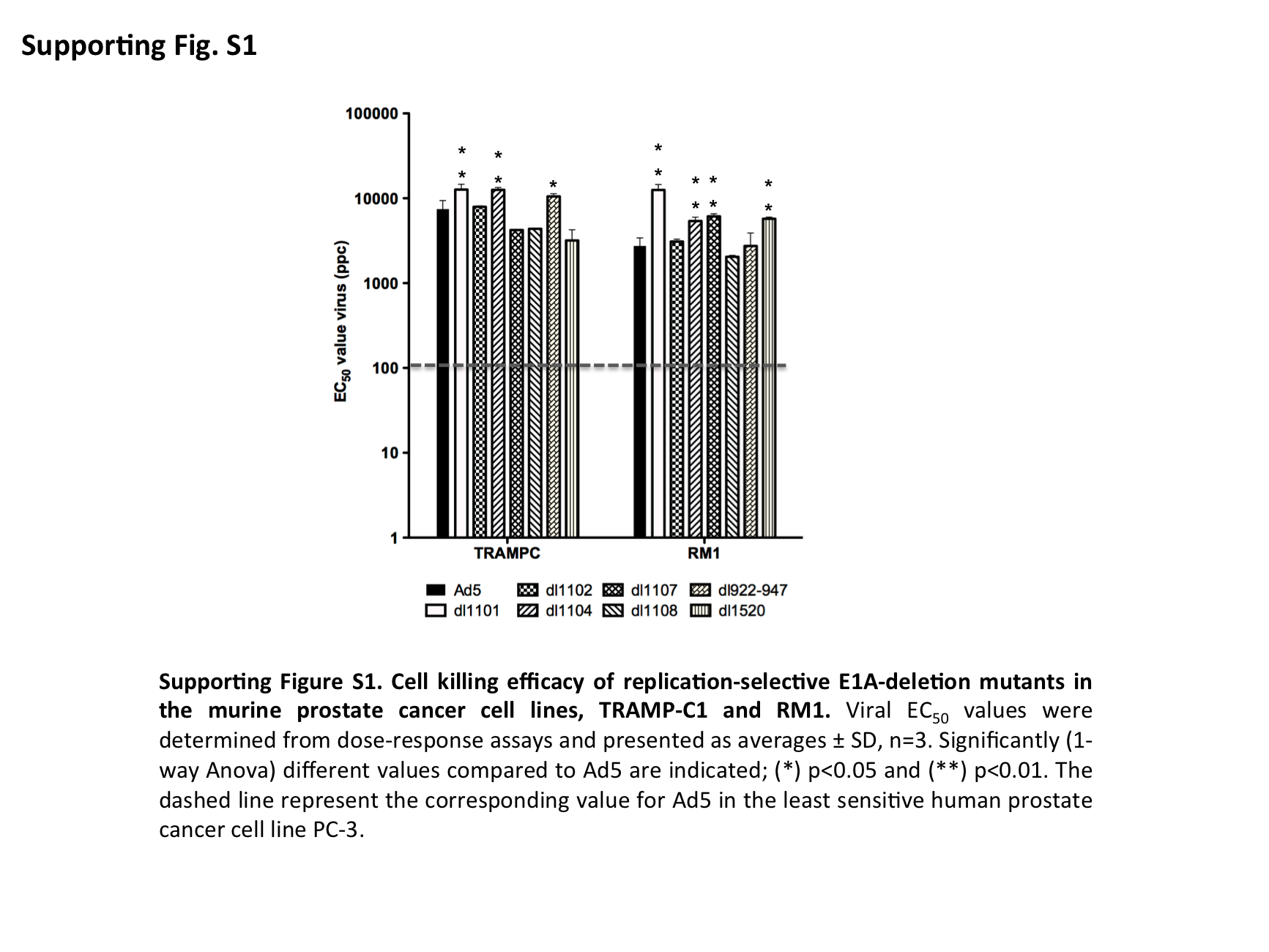

Supplement: Figure S1 — Cell killing efficacy of replication-selective E1A-deletion mutants in the murine prostate cancer cell lines, TRAMP-C1 and RM1. Viral EC50 values were determined from dose-response assays and presented as averages ± SD, n = 3. Significantly (1-way Anova) different values compared to Ad5 are indicated; (*) p<0.05 and (**) p<0.01. The dashed line represent the corresponding value for Ad5 in the least sensitive human prostate cancer cell line PC-3. (TIFF) [file pone.0046617.s001.tiff]

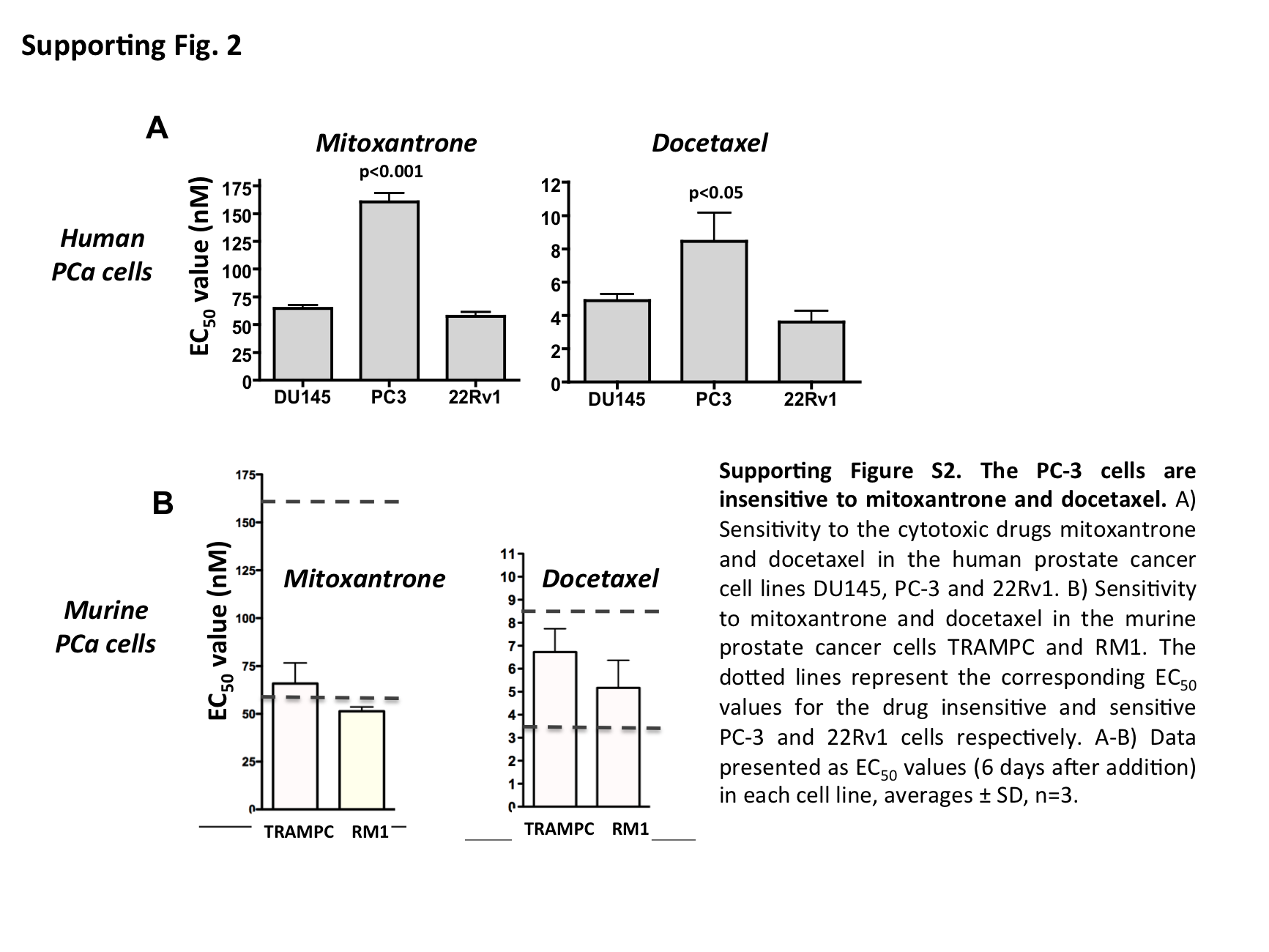

Supplement: Figure S2 — The PC-3 cells are insensitive to mitoxantrone and docetaxel. A) Sensitivity to the cytotoxic drugs mitoxantrone and docetaxel in the human prostate cancer cell lines DU145, PC-3 and 22Rv1. B) Sensitivity to mitoxantrone and docetaxel in the murine prostate cancer cells TRAMPC and RM1. The dotted lines represent the corresponding EC50 values for the drug insensitive and sensitive PC-3 and 22Rv1 cells respectively. A–B) Data presented as EC50 values (6 days after addition) in each cell line, averages ± SD, n = 3. (TIFF) [file pone.0046617.s002.tiff]

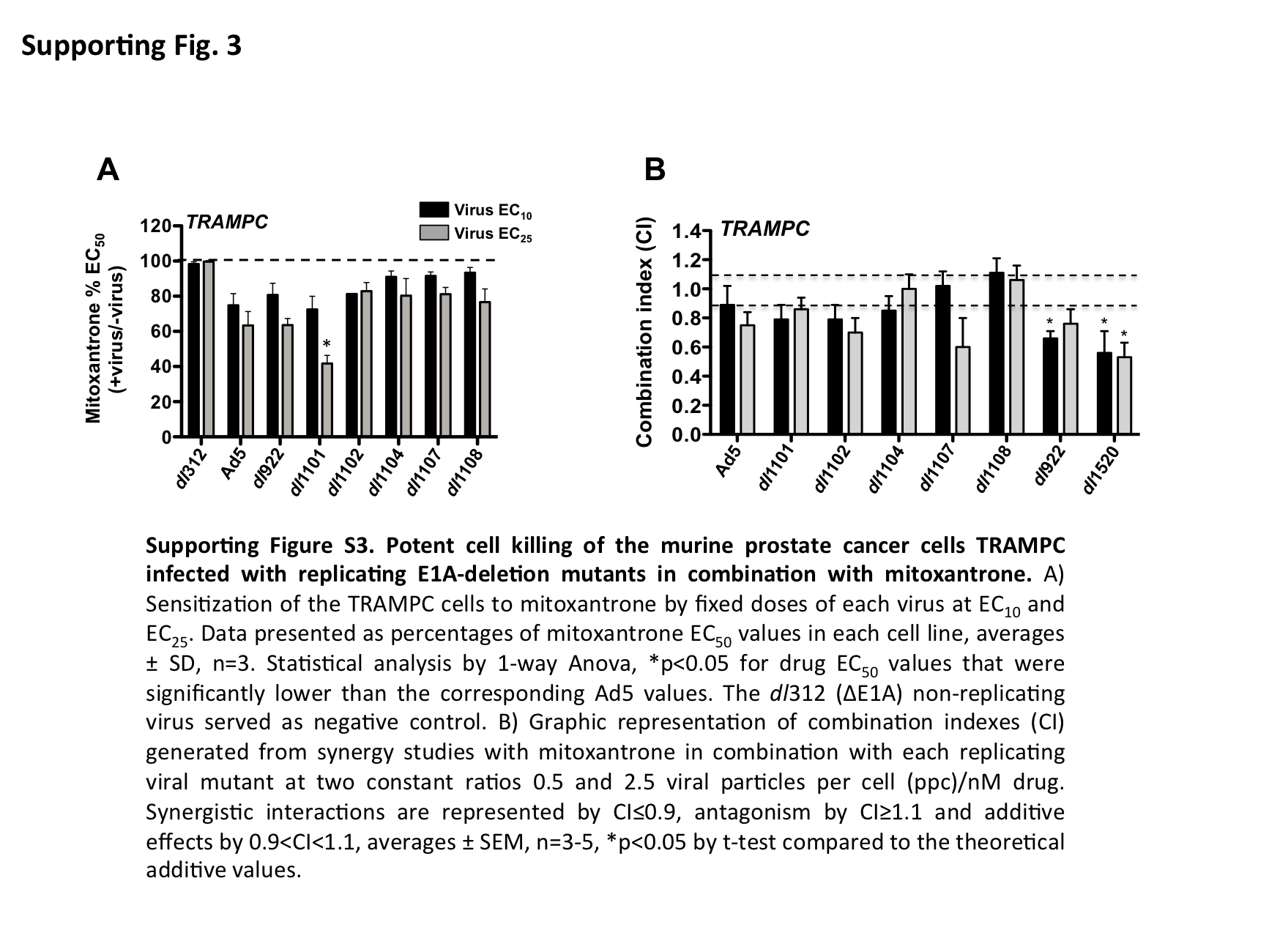

Supplement: Figure S3 — Potent cell killing of the murine prostate cancer cells TRAMPC infected with replicating E1A-deletion mutants in combination with mitoxantrone. A) Sensitization of the TRAMPC cells to mitoxantrone by fixed doses of each virus at EC10 and EC25. Data presented as percentages of mitoxantrone EC50 values in each cell line, averages ± SD, n = 3. Statistical analysis by 1-way Anova, *p<0.05 for drug EC50 values that were significantly lower than the corresponding Ad5 values. The dl312 (ΔE1A) non-replicating virus served as negative control. B) Graphic representation of combination indexes (CI) generated from synergy studies with mitoxantrone in combination with each replicating viral mutant at two constant ratios 0.5 and 2.5 viral particles per cell (ppc)/nM drug. Synergistic interactions are represented by CI≤0.9, antagonism by CI≥1.1 and additive effects by 0.9<CI<1.1, averages ± SEM, n = 3–5, *p<0.05 by t-test compared to the theoretical additive values. (TIFF) [file pone.0046617.s003.tiff]

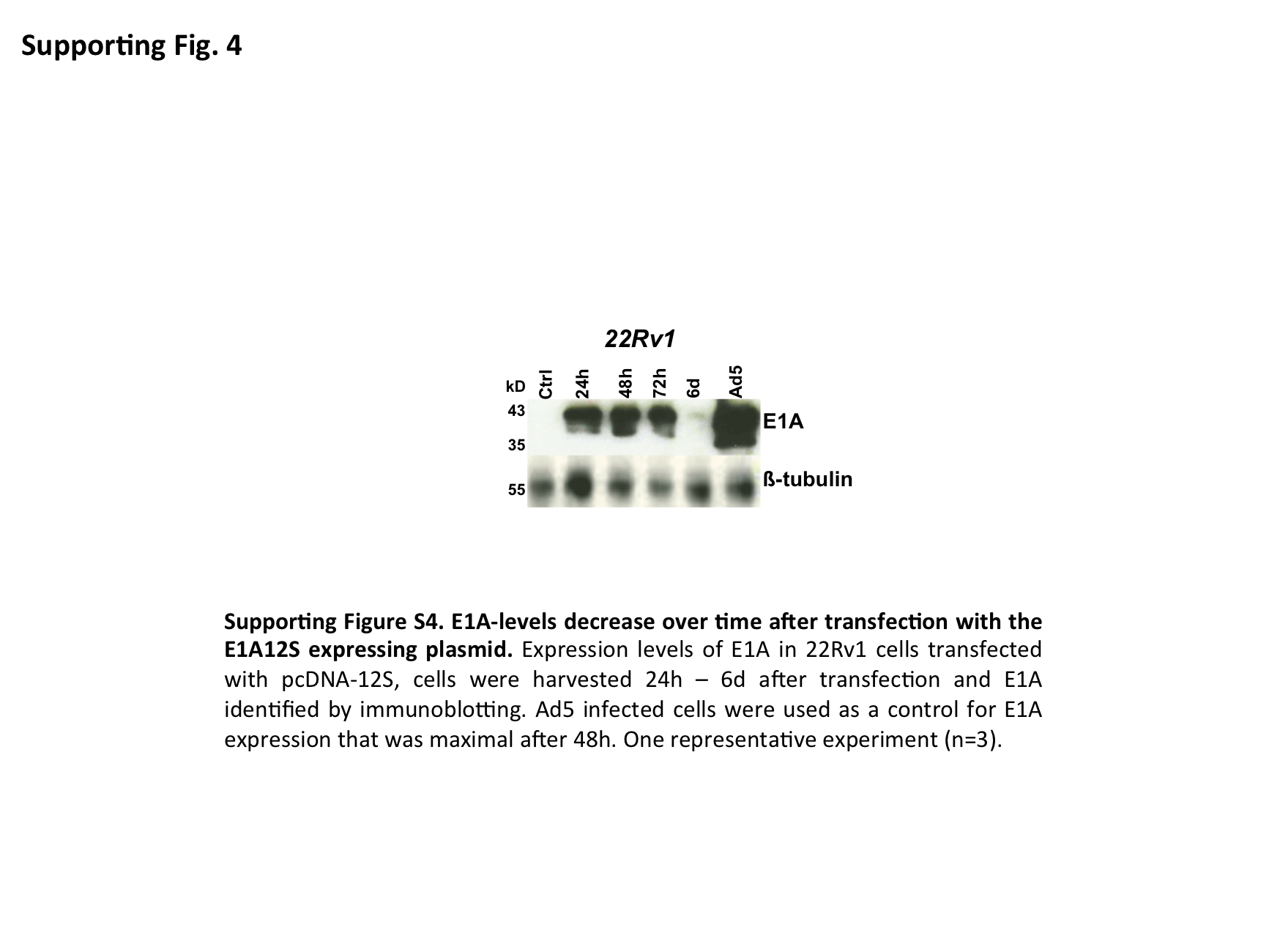

Supplement: Figure S4 — E1A-levels decrease over time after transfection with the E1A12S expressing plasmid. Expression levels of E1A in 22Rv1 cells transfected with pcDNA-12S, cells were harvested 24 h–6 d after transfection and E1A identified by immunoblotting. Ad5 infected cells were used as a control for E1A expression that was maximal after 48 h. One representative experiment (n = 3). (TIFF) [file pone.0046617.s004.tiff]

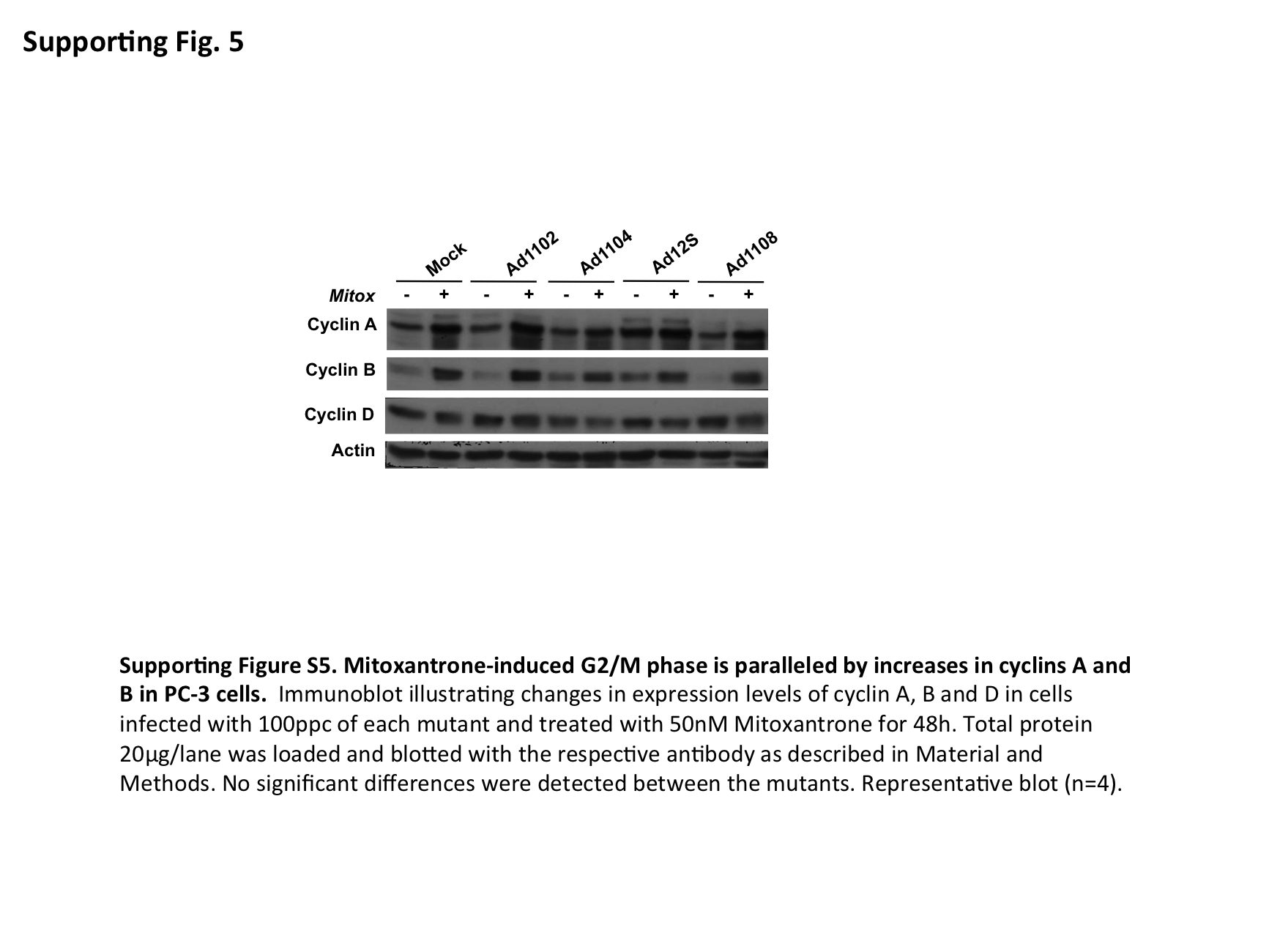

Supplement: Figure S5 — Mitoxantrone-induced G2/M phase is paralleled by increases in cyclins A and B in PC-3 cells. Immunoblot illustrating changes in expression levels of cyclin A, B and D in cells infected with 100 ppc of each mutant and treated with 50 nM Mitoxantrone for 48 h. Total protein 20 µg/lane was loaded and blotted with the respective antibody as described in Material and Methods. No significant differences were detected between the mutants. Representative blot (n = 4). (TIFF) [file pone.0046617.s005.tiff]
