# Supplementary material for: Adenovirus-Mediated Sensitization to the Cytotoxic Drugs Docetaxel and Mitoxantrone Is Dependent on Regulatory Domains in the E1ACR1 Gene-Region
Source: PLoS One. 2012 Oct 3;7(10):e46617. doi: 10.1371/journal.pone.0046617 (PMC3463540; doi:10.1371/journal.pone.0046617)
Supplement: Table S1 — Ratio of viral particle (vp) to replicating virus (pfu). (DOC) [file pone.0046617.s006.doc]

**Supporting Table S1. Ratio of viral particle (vp) to replicating virus (pfu).**

| Virus | vp/pfu * |
| --- | --- |
| Ad5 | 19.9 |
| AdE1A-12S | 34.5 |
| AdE1A-1102 | 39.2 |
| AdE1A-1104 | 18.9 |
| AdE1A-1108 | 26.5 |
| *dl*1101 | 15.5 |
| *dl*1102 | 13.4 |
| *dl*1104 | 9.1 |
| *dl*1108 | 13.8 |
| *dl*922-947 | 14.7 |
| *dl*1520 | 16.5 |

* pfu values obtained by TCID_50_ assays on HEK293 cells.
