# Supplementary material for: Adenovirus-Mediated Sensitization to the Cytotoxic Drugs Docetaxel and Mitoxantrone Is Dependent on Regulatory Domains in the E1ACR1 Gene-Region
Source: PLoS One. 2012 Oct 3;7(10):e46617. doi: 10.1371/journal.pone.0046617 (PMC3463540; doi:10.1371/journal.pone.0046617)
Supplement: Table S2 — Combination index (CI) for Ad5, AdE1A-12S or dl1520 in combination with mitoxantrone or docetaxel in the human prostate cancer cells PC3 and DU145. (DOC) [file pone.0046617.s007.doc]

**Supporting Table S2. Combination index (CI) for Ad5, AdE1A-12S or dl1520 in combination with mitoxantrone or docetaxel in the human prostate cancer cells PC3 and DU145.**

|  |  | **CI** | | | **CI** | | |
| --- | --- | --- | --- | --- | --- | --- | --- |
| ***Cells*** | ***Ratio (ppc/nM)*** | ***Ad5/M*** | ***Ad12S/M*** | ***dl1520/M*** | ***Ad5/D*** | ***Ad12S/D*** | ***dl1520/D*** |
| ***PC3*** | 0.5 | 0.6 | 0.8 | 0.3 | 0.7 | 0.9 | 0.9 |
|  | 2.5 | 0.4 | 0.6 | 0.3 | 0.8 | 0.8 | 0.9 |
|  | 12.5 | 0.9 | 0.5 | 0.3 | 0.9 | 0.7 | 0.7 |
|  | 62.5 | 1.1 | 0.5 | 0.5 | 1.1 | 0.5 | 0.8 |
| ***DU145*** | 0.5 | 0.7 | 0.6 | 0.6 | 0.8 | 0.7 | 0.7 |
|  | 2.5 | 0.7 | 0.5 | 0.5 | 1.0 | 0.6 | 0.6 |
|  | 12.5 | 0.9 | 0.7 | 0.7 | 0.9 | 0.8 | 0.7 |
|  | 62.5 | 1.1 | 0.8 | 0.8 | 0.9 | 0.7 | 0.9 |

Representative data from 3-4 studies.
